# Fluorescent protein-scorpion toxin chimera is a convenient molecular tool for studies of potassium channels

**DOI:** 10.1038/srep33314

**Published:** 2016-09-21

**Authors:** Alexey I. Kuzmenkov, Oksana V. Nekrasova, Kseniya S. Kudryashova, Steve Peigneur, Jan Tytgat, Alexey V. Stepanov, Mikhail P. Kirpichnikov, Eugene V. Grishin, Alexey V. Feofanov, Alexander A. Vassilevski

**Affiliations:** 1 Shemyakin-Ovchinnikov Institute of Bioorganic Chemistry, Russian Academy of Sciences, Moscow, Russia; 2 Biological Faculty, Lomonosov Moscow State University, Moscow, Russia; 3 ICV Ltd, Moscow, Russia; 4 Laboratory of Toxicology and Pharmacology, University of Leuven, Leuven, Belgium

## Abstract

Ion channels play a central role in a host of physiological and pathological processes and are the second largest target for existing drugs. There is an increasing need for reliable tools to detect and visualize particular ion channels, but existing solutions suffer from a number of limitations such as high price, poor specificity, and complicated protocols. As an alternative, we produced recombinant chimeric constructs (FP-Tx) consisting of fluorescent proteins (FP) fused with potassium channel toxins from scorpion venom (Tx). In particular, we used two FP, eGFP and TagRFP, and two Tx, OSK1 and AgTx2, to create eGFP-OSK1 and RFP-AgTx2. We show that these chimeras largely retain the high affinity of natural toxins and display selectivity to particular ion channel subtypes. FP-Tx are displaced by other potassium channel blockers and can be used as an imaging tool in ion channel ligand screening setups. We believe FP-Tx chimeras represent a new efficient molecular tool for neurobiology.

Ion channels are proteins that set the permeability and control ion fluxes across cell membranes. Found ubiquitously, they play central roles in many physiological processes ranging from simple taxis in unicellular organisms to processing information in the brain of higher primates, and are believed to represent one of the core features of life^1^. In the human genome, over 200 genes are known to encode functional subunits of diverse ion channels, which may assemble into homo- and hetero-oligomers providing a vast multiplicity of these proteins^2^,^3^. Ion channels are the third most numerous group of proteins involved in signal transduction^4^ and the second largest target for existing drugs^5^. However, they are also considered highly underexploited in terms of drug discovery^6^, and many more compounds affecting ion channels are anticipated to enter the pipeline.

Traditionally, research into ion channel structure and function has relied considerably on the application of natural toxins as powerful and selective molecular tools. Many of these compounds demonstrate unmatched properties: they are able to bind to ion channels with nanomolar or picomolar affinity, discriminate between very similar ion channel varieties, and alter channel function in desired ways: block, activate, potentiate, change kinetics, etc.^7^,^8^,^9^,^10^. For example, sodium channel proteins were purified due to the use of tetrodotoxin and scorpion α-toxins^11^. Likewise, snake α-neurotoxins helped to isolate acetylcholine receptors^12^. Current classification of calcium channels is based on their sensitivity to spider and cone snail toxins, which are routinely used to differentiate these channels in neurobiology today^13^. In structural biology, toxins are widely used to capture ion channels in different functional states, such as open, closed or inactivated^14^,^15^,^16^,^17^.

Here we follow the trend of toxin application to the study of ion channels. We present a simple and robust approach that will substantially enlarge the neurobiology research toolkit. We produced chimeric molecules (FP-Tx) that combined two functional parts: fluorescent proteins (FP) and scorpion potassium channel toxins (Tx). The Tx part conferred nanomolar affinity and selectivity to particular potassium channel isoforms, as was shown directly on recombinant ion channel preparations and by electrophysiological measurements. The FP part served as a molecular beacon to detect the chimeras and hence the target ion channels by fluorescence microscopy. We went on to demonstrate the applicability of the new tools, for example, to screen new ligands of ion channels. Previous alternatives to channel labeling were to use either chemically modified toxins or antibodies. Both possibilities suffered from serious drawbacks, such as complicated synthesis and high price (chemical modification), and poor selectivity and need to work with fixed samples (antibodies). We rationalize that our FP-Tx tools are easy to use and can be produced just by the recombinant technique, avoiding any chemical modification. As an outlook, we suggest that molecules with similar design will find successful application throughout biochemistry and biotechnology.

## Results

### Fluorescent protein-scorpion toxin (FP-Tx) chimeras

**Figure 1:**
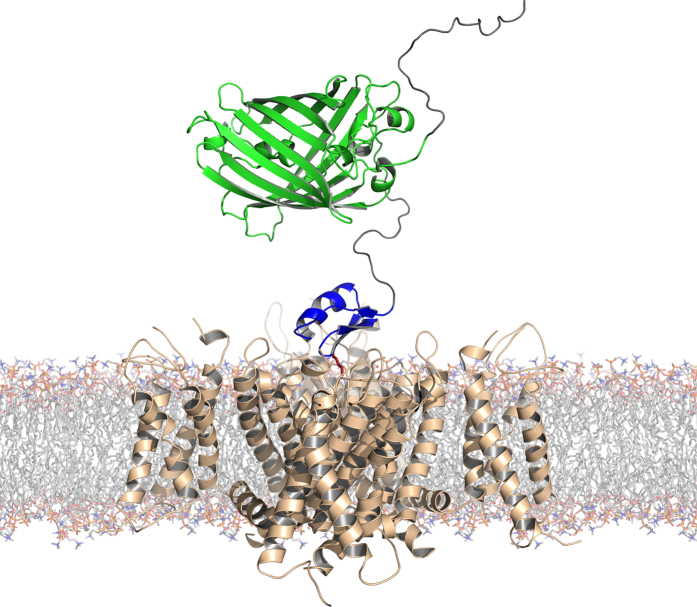
Molecular model of complex formed between eGFP-OSK1 and K_V_. FP-Tx modules are colored: eGFP is in green, and OSK1 is in blue; flexible N-terminus and linker are in gray. The functionally crucial lysine residue of Tx is shown in red pointing to the channel selectivity filter. The model was built based on homology with the known structures of eGFP (PDB ID: 2Y0G), OSK1 (1SCO), and complex of the K_V_1.2-K_V_2.1 paddle chimera with charybdotoxin (4JTA).

We designed two expression cassettes encoding Tx fused to FP *via* flexible and hydrolysable linker sequences (see Fig. 1 for overall scheme, Suppl. Fig. 1 for gene structures, and Suppl. Information for details). We selected two well studied Tx, OSK1 and AgTx2, from venoms of scorpions *Orthochirus scrobiculosus* and *Leiurus quinquestriatus hebraeus*, respectively. These Tx are pore blockers of voltage-gated potassium channels (K_V_) that act at (sub)nanomolar concentrations: OSK1 blocks efficiently K_V_1.1, K_V_1.2, and K_V_1.3 channel isoforms and displays moderate affinity to the calcium-activated potassium channel K_Ca_3.1^18^, while AgTx2 inhibits K_V_1.1, K_V_1.2, K_V_1.3, and K_V_1.6^19^. We also selected two FP: eGFP and TagRFP. Both FP are characterized by high brightness and ability to retain monomeric form even at high concentrations, and are widely used as tags in cellular and animal studies.

**Figure 2:**
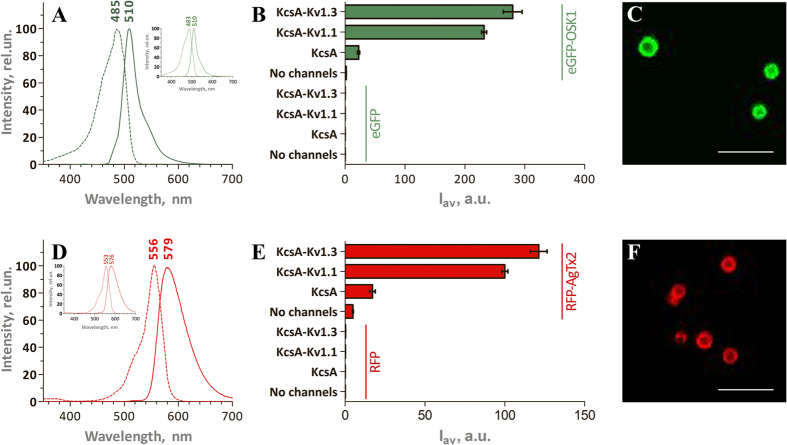
FP-Tx fluorescence and binding to KcsA-K_V_ hybrid channels. (**A,D**) Fluorescence excitation (dashed lines) and emission (solid lines) spectra of eGFP-OSK1 (**A**) and RFP-AgTx2 (**D**). Insets show excitation and emission spectra of separate eGFP and TagRFP. (**B**,**E**) Relative binding of eGFP-OSK1 (20 nM) and eGFP (20 nM) (**B**), and RFP-AgTx2 (10 nM) and RFP (10 nM) (**E**) to KcsA-KV1.3, KcsA-KV1.1, and KcsA-bearing spheroplasts and to spheroplasts without recombinant channels. (**C,F**) Typical confocal fluorescence images of KcsA-K_V_1.3-bearing spheroplasts stained with eGFP-OSK1 (**C**) and KcsA-K_V_1.1-bearing spheroplasts stained with RFP-AgTx2 (**F**). The length of the scale bars is 4 μm for both images.

eGFP-OSK1 and RFP-AgTx2 chimeras were isolated from *E. coli* lysates by affinity chromatography and purified by size-exclusion chromatography (Suppl. Fig. 2A). SDS-PAGE, selective proteolysis and MALDI MS (Suppl. Fig. 2B) were used to check correct protein production and purification. Fluorescence excitation and emission spectra of eGFP-OSK1 and RFP-AgTx2 (Fig. 2A,D) are identical to native eGFP and TagRFP, respectively. The yields of recombinant eGFP-OSK1 and RFP-AgTx2 were high, reaching 100 mg per 1 liter of bacterial culture.

### Electrophysiology

**Figure 3:**
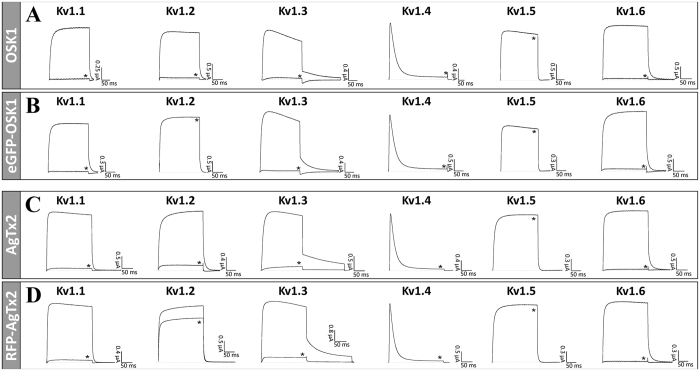
FP-Tx activity studied by electrophysiology. Shown are representative traces of currents through K_V_1.1-1.6 channels in control and after application of 1 μM OSK1 (**A**), and 1 μM eGFP-OSK1 (**B**), or 0.5 μM AgTx2 (**C**), and 0.5 μM RFP-AgTx2 (**D**) (indicated with asterisks).

**Figure 4:**
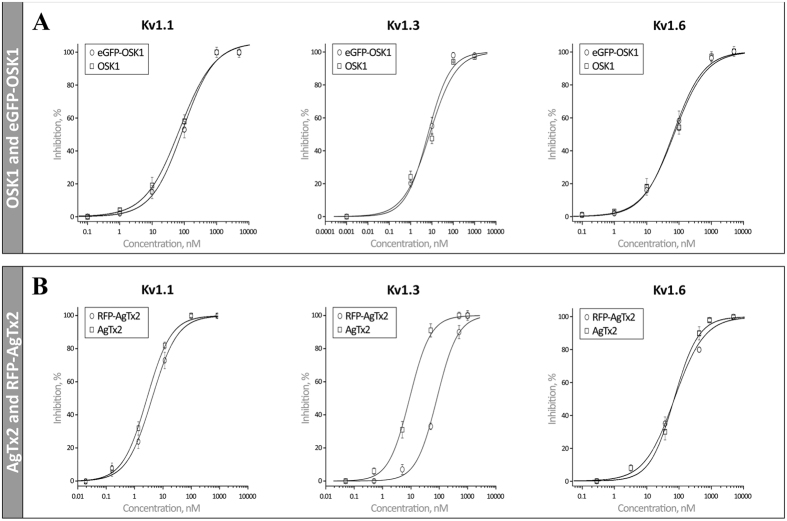
Concentration-response curves for FP-Tx on K_V_1 channels. Comparison between OSK1 and eGFP-OSK1 (**A**), or AgTx2 and RFP-AgTx2 (**B**).

Two-electrode voltage clamp technique was used to check the activity of FP-Tx chimeras on K_V_ channels. Figure 3 shows the induced inhibition of K_V_1.1–1.6 channels after application of FP-Tx and native OSK1 and AgTx2. Importantly, no activity was seen on K_V_1.4 and K_V_1.5 channels for either native Tx or FP-Tx chimeras. Quite unexpectedly, we observed a loss of activity against K_V_1.2 for both FP-Tx chimeras. Another new finding is the considerably high activity of OSK1 against K_V_1.6 (Figs 3 and 4), which revises the previously reported data on the absence of such activity^18^. A similar block of K_V_1.6 is observed for eGFP-OSK1 (Figs 3 and 4). The reason of this discrepancy with earlier studies is unclear but may in part be due to the different experimental setups (i.e. voltage clamp in oocytes and patch clamp in mammalian cells).

Concentration-response curves were constructed for both chimeras and native Tx on K_V_1.1, 1.3, and 1.6 channels (Fig. 4). For OSK1 and eGFP-OSK1 we observed the following IC_50_ values (mean ± S.E., n ≥ 3): 72 ± 14 and 90 ± 17 nM (against K_V_1.1), 6 ± 2 and 8 ± 2 nM (K_V_1.3), and 71 ± 11 and 66 ± 5 nM (K_V_1.6). And for AgTx2 and RFP-AgTx2 the IC_50_ values were (mean ± S.E., n ≥ 3): 2 ± 0.1 and 3 ± 0.4 nM (K_V_1.1), 9 ± 2 and 83 ± 13 nM (K_V_1.3), and 92 ± 10 and 94 ± 16 nM (K_V_1.6). Hill coefficients are reported in Table 1.

### Hybrid KcsA-K_V_1 channels

Fluorescent systems based on bacterial expression of hybrid KcsA-K_V_1 channels, in which the extracellular loop of bacterial potassium channel KcsA is substituted with corresponding regions from K_V_ represent a robust method to screen novel K_V_ ligands^20^,^21^,^22^. Such hybrids bind specifically pore blockers of K_V_, and the dissociation constants of these complexes are in good agreement with the constants estimated from electrophysiological measurements on native K_V_ channels.

**Figure 5:**
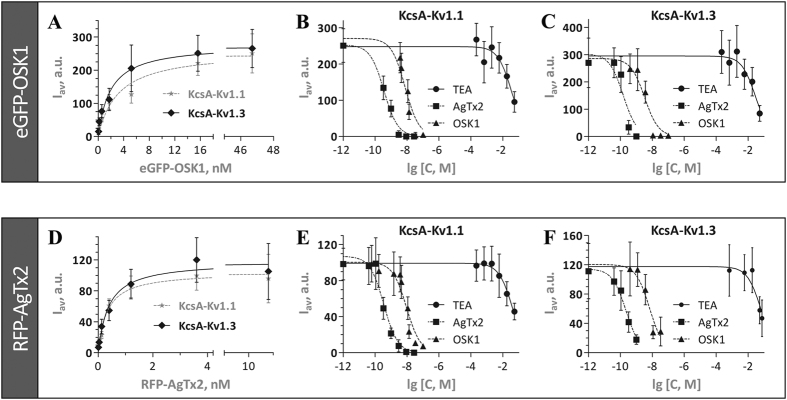
Binding of FP-Tx to KcsA-K_V_1-bearing spheroplasts and their displacement by different ligands. **(A,D)** Saturation curves of eGFP-OSK1 and RFP-AgTx2 binding to KcsA-K_V_1.1 or KcsA-K_V_1.3-bearing spheroplasts. **(B,C)** Competition between eGFP-OSK1 and different ligands for the binding to KcsA-K_V_1.1 or KcsA-K_V_1.3-bearing spheroplasts. **(E,F)** Competition between RFP-AgTx2 and different ligands for the binding to KcsA-K_V_1.1 or KcsA-K_V_1.3-bearing spheroplasts. Data of representative experiments are shown. Mean ± S.E. values are presented (number of cells per point, n ≥ 150).

We utilized a system based on KcsA-K_V_1.1 and KcsA-K_V_1.3 hybrids expressed in *E. coli* to characterize the mode of eGFP-OSK1 and RFP-AgTx2 interaction with K_V_. Both FP-Tx chimeras were able to bind to *E. coli* spheroplasts bearing hybrid channels (Fig. 2B,E) and fluoresce brightly when bound, thus facilitating fluorescent imaging (Fig. 2C,F). Both chimeras demonstrate a concentration-dependent binding with saturation (Fig. 5A,D) and do not bind to spheroplasts without hybrid channels. eGFP-OSK1 and RFP-AgTx2 bind to KcsA-Kv1.1 with dissociation constants (mean ± S.E., n = 3) of 3.2 ± 1.1 and 0.4 ± 0.1 nM, respectively. The dissociation constants for complexes of eGFP-OSK1 and RFP-AgTx2 with KcsA-Kv1.3 are 1.9 ± 0.4 and 0.38 ± 0.09 nM (mean ± S.E., n = 3). The binding is specific and reversible: FP-Tx are displaced from the complexes by pore blockers of K_V_ including OSK1, AgTx2, and TEA (Fig. 5B,C,E,F and Suppl. Table 2). As expected, competition between non-labeled Tx and FP-Tx occurs in the nanomolar concentration range, and the apparent dissociation constants of Tx calculated from competitive binding experiments are similar to those published previously^18^,^19^. TEA, a non-specific small organic pore blocker of K_V_, demonstrates typical activity at millimolar concentrations. The apparent dissociation constants of Tx determined versus either FP-Tx are nearly equal (Suppl. Table 2), confirming an identical binding site for both chimeras on K_V_1.

**Figure 6:**
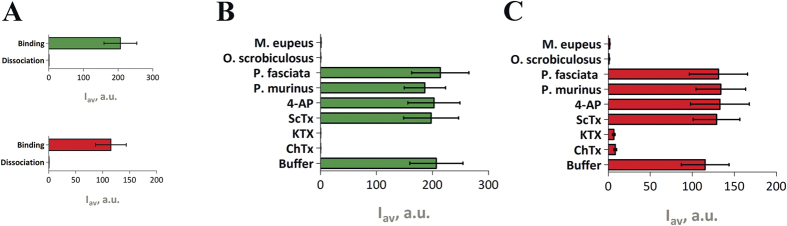
Application of FP-Tx in spheroplast binding assay as a screening tool. (**A**) Reversibility of eGFP-OSK1 and RFP-AgTx2 binding to KcsA-K_V_1.3 on spheroplast membrane. The “binding” bar shows signal from spheroplasts incubated with eGFP-OSK1 or RFP-AgTx2 (5.6 nM FP-Tx, 2 h, 37 °C), and the “dissociation” bar shows signal from the same spheroplasts incubated in a ligand-free medium for 4 h for FP-Tx wash-out. (**B,C**) Influence of potassium channel blockers charybdotoxin (ChTx, 300 nM), kaliotoxin (KTX, 30 nM), scyllatoxin (ScTx, 1 μM), and 4-aminopyridine (4-AP, 10 mM) and crude venoms (38 μg/ml) of *M. eupeus*, *O. scrobiculosus*, *P. fasciata*, and *P. murinus* on the binding of 5.6 nM eGFP-OSK1 (**B**) and RFP-AgTx2 (**C**) to KcsA-K_V_1.3-bearing spheroplasts. Mean ± S.E. values are shown (n = 3).

To demonstrate that FP-Tx can be used for ligand screening in the spheroplast binding assay, we measured the influence of different potassium channel blockers and crude animal venoms on the binding of FP-Tx to KcsA-K_V_1.3-bearing spheroplasts (Fig. 6). Displacement of FP-Tx from complexes with KcsA-K_V_1.3 was considered as an ability to recognize the presence of pore blockers in the added solution. Exactly as expected, K_V_1 blockers charybdotoxin (ChTx)^19^ and kaliotoxin (KTX)^23^ competed with FP-Tx for the binding to KcsA-Kv1.3-bearing spheroplasts, while scyllatoxin (ScTx)^24^, a blocker of calcium-activated potassium channels, and 4-aminopyridine (4-AP), a small-molecule blocker that binds to the inner cavity of K_V_1^25^, did not. Four crude animal venoms were chosen in order to show that FP-Tx are able to report presence of K_V_1.3 pore blockers in complex biological mixtures. Indeed, eGFP-OSK1 and RFP-AgTx2 were displaced from complexes with KcsA-K_V_1.3 by scorpion (the lesser Asian scorpion *Mesobuthus eupeus* and Central Asian scorpion *Orthochirus scrobiculosus*) venoms containing potassium channel blockers but not by spider (the Sri Lanka ornamental tarantula *Poecilotheria fasciata* and baboon spider *Pterinochilus murinus*) venoms lacking such compounds (Fig. 6).

## Discussion

Potassium channels are the most widespread and diverse ion channels. A short list of their functions includes setting the resting potential, regulation of the action potential, ion transport, neuronal transmission, cell proliferation, and cell-cell communication. It is not surprising that potassium channels are involved in many pathological states and represent important targets for drug discovery^26^. For example, ATP-sensitive potassium channels (K_ATP_) regulate insulin secretion by pancreatic islet cells and mutations in the channel genes can cause neonatal diabetes mellitus^27^. The hERG channel is responsible for a repolarizing current in the cardiac action potential, and mutations in its gene may lead to fatal cardiac arrhythmias^28^. The voltage-gated K_V_1.3 channel plays a pivotal role in T cell activation and represents a therapeutic target for treatment of autoimmune diseases^29^.

Localization and visualization of ion channels in cells and tissues is of high importance to the progress of both the fundamental and applied research. To date, investigators have to rely on a limited set of tools, each with some important shortcomings. The introduction of FP-Tx chimeras may address the need for the simpler solutions to ion channel visualization.

Natural neurotoxins target particular ion channel subtypes with high affinity and specificity and may be used as precision research tools^30^. Probably the best example is potassium channel pore blockers extracted from scorpion venom^8^. Figure 1 presents our strategy involving FP-Tx schematically. A recent crystal structure of complex between the classic ChTx and voltage-gated potassium channel^14^ suggests that the N-terminus can be modified without seriously affecting the channel-blocking activity. This is why we attached an FP through a flexible linker to the N-termini of OSK1 and AgTx2. The resulting chimeras largely retain the high affinity and specificity to K_V_1 (Figs 3, 4 and 5, Table 1). However, we also see certain limitations (both chimeras lost activity on K_V_1.2, and RFP-AgTx2 was less active against K_V_1.3 than unmodified AgTx2), and it is therefore advised to check the specificity profile of each new FP-Tx. Due to the modified selectivity profiles, both chimeras may find application for K_V_1.1 and K_V_1.2 homotetramer discrimination, for example. FP-Tx chimeras can substitute ligands of ion channels labeled with organic fluorophores in traditional applications.

Tagged ligands can be successfully used in analytical systems designed for screening of novel ion channel modulators. For such tasks the fluorescent systems are a powerful alternative to radioligand and patch-clamp techniques. It has been previously demonstrated that rhodamine-labeled AgTx2 can be used in screening setups directed on novel potassium channel blockers discovery^21^,^22^. We show here that both eGFP-OSK1 and RFP-AgTx2 can be effectively used instead. These FP-Tx chimeras bind selectively to *E. coli* spheroplasts expressing cognate potassium channels (Fig. 2B,C,E,F). They also show suitable dissociation constant values and are displaced by classic potassium channel pore blockers (Fig. 5 and Suppl. Table 2).

Electrophysiology is considered the “gold standard” in ion channel research. However, it requires specialized equipment and sophisticated skills, and is time-consuming. Radioligand binding assay was successfully used in early quantitative experiments on ligand-receptor interactions, but currently only a limited number of scientific groups keep this technique in their arsenal due to safety limitations. In recent years the focus has shifted towards detection of fluorescence as a mainstream technique, such as in flow cytometry, single molecule fluorescent microscopy, and confocal microscopy.

Until now fluorescent labeling of peptide ligands was performed by one of the two ways. (1) Attachment of a fluorophore to the N-terminal amino group of the peptide at the last step of solid phase peptide synthesis. This option is mainly available through expensive commercial peptide synthesis services. (2) Conjugation of the peptide with a reactive organic label. Most often the chemistry involves a thiol group of an additionally introduced cysteine residue or an amino group (N-terminal or in lysine residues). In this case, labeling is usually compromised by a low reaction yield, multiple sites of labeling, and considerable product loss during separation of different variants of conjugates. Introduction of high-precision click chemistry protocols has largely overcome this deficiency^31^. Another major bottleneck is loss of activity due to modification, a fluorophore-specific effect, which is almost impossible to predict. This makes it virtually impossible to choose beforehand which labeling strategy to use to produce a fluorescent ligand with the desired qualities^32^.

Utilization of recombinant FP-Tx resolves this difficulty. The modification position of the FP moiety is strictly defined. The protein tag, in spite of its large size, affects moderately the properties of the Tx module. We have demonstrated that different Tx modules can be successfully paired with different FP modules. A flexible linker between the modules seems to be essential, but according to our results its structure and length can vary widely. It is 15-residues-long (G_4_S)_3_ in eGFP-OSK1, and 50-residues-long and contains a His-Tag in RFP-AgTx2 (see Suppl. Information). Additional functional modules like specific enzyme cleavage sites can be introduced into the linker for special purposes. A high yield of recombinant FP-Tx production (up to 100 mg per liter of bacterial culture), correct folding and simplicity of purification reduce drastically the cost of the fluorescent ligands and extend their availability for research laboratories. One foreseen restriction may be recombinant production of the Tx moiety given the usually high number of disulfide bridges in neurotoxins, but the state-of-the-art techniques ensure correct folding of even very hard-expressing proteins. Expression in yeast, insect or mammalian cells, or cell-free systems is viewed as a feasible alternative to our bacterial expression protocols.

We see no obstacle to a much wider realization of the proposed concept. Over 250 potassium channel blockers from scorpion venom are currently known, and some of them display marked channel subtype specificity^33^,^34^. These specific Tx are the primary candidates for further FP-Tx engineering. The obvious limitation of the FP-Tx reported here is the rather wide profile with respect to different ion channel isoforms. Focus on more specific ligands is a necessary future step forward. Another important point is that while experiments *in vitro* are often performed with homomeric channels, heteromers are prevalent *in vivo*^35^. To rigorously validate the specificity profile of any new tool one should thereby include heteromeric channels.

In addition, the known 3D structures of complexes formed between natural toxins and ion channels suggest that in the following cases the N-termini of toxins point away from the interaction surface and may be used for FP attachment: snake α-neurotoxins to label acetylcholine receptors^36^; spider toxin PcTx1 to label acid-sensing ion channels^17^; and Con-ikot-ikot, a cone snail toxin to label glutamate receptors^16^. The existing models of scorpion α and β-toxins in complex with sodium channels^37^,^38^ also predict a free N-terminus; these toxins are able to discriminate between otherwise very similar ion channels. Alternatively, the positioning of FP and Tx elements may be reversed, if it is the C-terminus of toxins that can be modified.

On the other hand, there is a great variety of FP adopted for use in practice^39^. We anticipate a great diversity of FP-Tx chimeras ensuring visualization of multiple ion channels simultaneously by using different colors, and providing means to detect co-localization by FRET or similar techniques. The diversity will arise from the available variety of both Tx and FP modules and the apparently unlimited possibility to produce any combination of the two.

## Methods

### Gene design, synthesis, and cloning

Oligonucleotide primers used for cloning are shown in Suppl. Table 1. A chimeric gene encoding eGFP-OSK1 was generated by overlap extension PCR that joined two fragments. The first fragment comprising the eGFP gene and a 3′-flanking linker sequence was PCR-amplified from the plasmid pUC-eGFP with eGFP-f and a linker-specific eGFP-r primers. The second fragment encoding OSK1 was amplified from the plasmid pET-32b-OSK1 with OSK1-f and OSK1-r primers. The entire coding sequence was cloned into pET-28a (Novagen) at NdeI/EcoRI sites to yield the pET-28a-eGFP-OSK1 expression vector. The pET-28a-eGFP vector was obtained by cloning the eGFP gene alone, which was amplified with the eGFP-f and eGFP-s primers.

A chimeric gene encoding RFP-AgTx2 was generated by three steps. First, the TagRFP gene was PCR-amplified from pTagRFP-C (Evrogen, Russia) with primers RFP-f and RFP-r and cloned into pET-23d (Novagen) at NcoI/EcoRI sites yielding pET-23d-RFP. EcoRI/HindIII sites of this vector were used to clone a linker sequence derived from pET-32b (Novagen) by amplification of a DNA fragment downstream of the *trx* gene with primers L-f and L-r, yielding pET-23d-RFP-L1. DNA fragment encoding AgTx2 fused at its 5′-terminus with a sequence encoding a TEV protease cleavage site was obtained by PCR using a set of primers (AgTx-f1, -f2, and AgTx-r1, -r2) and cloned into pET-23d-RFP-L1 at KpnI/HindIII sites yielding pET-23d-RFP-AgTx2. Correct cloning of target genes was confirmed by sequencing.

### Expression and purification

*E. coli* BL21(DE3) cells transformed with pET-28a-eGFP-OSK1 were cultured at 37 °C in LB medium in the presence of 15 μg/ml kanamycin to the mid-log phase. Expression of eGFP-OSK1 was induced by 0.5 mM IPTG and the culture was further incubated at 25 °C for 20 h. Cells were harvested by centrifugation, disrupted by sonication, and the chimeric eGFP-OSK1 protein was purified from the soluble fraction by affinity chromatography on a TALON Superflow resin (Clontech) following the manufacturer’s protocol. Further purification was performed by size-exclusion chromatography on a TSK 2000SW column (7.5 × 600 mm, 12.5 nm pore size, 10 μm particle size; Toyo Soda Manufacturing) in PBS, pH 7.4. The same procedures were carried out for eGFP.

Cultivation of *E. coli* Rosetta-gami(DE3)pLysS harboring the pET23-RFP-AgTx2 plasmid was carried out in TB medium containing 100 μg/ml ampicillin, 15 μg/ml kanamycin, 12.5 μg/ml tetracycline, and 34 μg/ml chloramphenicol at 37 °C. RFP-AgTx2 expression was induced with 0.1 mM IPTG, and cultivation was continued at 18 °C for 20 h.

Expression and purification of the target proteins was followed by SDS-PAGE. Concentration of the final FP-Tx preparations was measured by absorption spectroscopy and calculated using ε_489_ = 55000 M^−1^cm^−1^ for eGFP-OSK1 and ε_555_ = 100000 M^−1^cm^−1^ for RFP-AgTx2.

### Selective proteolysis

Cleavage of chimeric proteins into separate modules (FP and Tx) was carried out by endoproteinase Glu-C (Roche) for eGFP-OSK1 and by TEV protease (Sigma-Aldrich) for RFP-AgTx2 following the manufacturers’ guidelines. Digestion products were separated by reversed-phase HPLC.

### Mass spectrometry

Molecular masses of compounds were measured on an Ultraflex TOF-TOF (Bruker Daltonik GmbH) spectrometer by the staff of laboratory of proteomics, Shemyakin-Ovchinnikov Institute of Bioorganic Chemistry.

### Fluorescence spectroscopy

Absorption, fluorescence excitation and emission spectra of 0.5 μM FP-Tx were measured in 10 mM Tris-HCl, pH 7.5. A Cary Eclipse instrument (Varian) was used.

### Molecular modeling

A model of eGFP-OSK1 was created using the UCSF Chimera 1.10.1 interface^40^ to Modeller 9.14^41^, using the structure of eGFP (PDB ID: 2Y0G) and OSK1 (1SCO) as templates. The N-terminal region containing the His-tag and the (SG_4_)_3_ linker between the eGFP and OSK1 modules were presented as disordered.

3D alignment of OSK1 from the generated eGFP-OSK1 model and ChTx from the spatial structure of its complex with the K_V_1.2-K_V_2.1 paddle chimera (4JTA) was performed using PyMOL 1.7.4 (Schrödinger).

### Hybrid KscA-K_V_ channels

*E. coli* BL21(DE3) cells that express KcsA-K_V_1.1 or KcsA-K_V_1.3 hybrid channels in the inner membrane were cultivated and spheroplasts were prepared as described elsewhere^21^,^22^. For binding experiments, KcsA-K_V_1.1 or KcsA-K_V_1.3-presenting spheroplasts (1000 cells μL^−1^) were incubated for 2 h at 37 °C with increasing concentrations of eGFP-OSK1 or RFP-AgTx2 in a buffer containing 10 mM Tris-HCl (pH 7.5), 0.25 M sucrose, 0.3 mM EDTA, 4 mM KCl, 10 mM MgCl_2_, and 0.1% BSA. For competitive binding, the spheroplasts were incubated with eGFP-OSK1 (14 nM for KcsA-Kv1.1 and 7 nM for KcsA-Kv1.3) or RFP-AgTx2 (2.43 nM) and increasing concentrations of non-labeled K_V_ pore blockers (4-AP, AgTx2, ChTx, KTX, OSK1, ScTx, or TEA) for 2 h at 37 °C. Labeled and non-labeled ligands were added simultaneously to the spheroplasts.

### Expression of K_V_ in *Xenopus laevis* oocytes

For the expression of mammalian voltage-gated potassium channels K_V_1.1–1.6 (all from rat except for human K_V_1.3) in *Xenopus* oocytes, all steps were performed as described elsewhere^21^,^42^. Linearized plasmids harboring K_V_ genes were transcribed using the T7 or SP6 mMESSAGE-mMACHINE transcription kit (Ambion). The harvesting of stage V-VI oocytes from an anaesthetized female *Xenopus laevis* frog was done as previously described^42^; KU Leuven guidelines for animal welfare were followed. Oocytes were injected with 50 nl of cRNA at a concentration of 1 ng/nl using a micro-injector (Drummond Scientific). The oocytes were incubated in ND96 solution containing 96 mM NaCl, 2 mM KCl, 1.8 mM CaCl_2_, 2 mM MgCl_2_, and 5 mM HEPES (pH 7.4), supplemented with 50 mg/L gentamycin sulfate.

### Electrophysiological recordings

Two-electrode voltage-clamp recordings were performed as described^21^,^42^ at room temperature (18–22 °C) using a Geneclamp 500 amplifier (Molecular Devices) controlled by a pClamp data acquisition system (Axon Instruments). Whole-cell currents from oocytes were recorded 1–4 days after injection. Bath solution was ND96. Voltage and current electrodes were filled with 3 M KCl. Resistances of both electrodes were kept between 0.7 and 1.5 MΩ. The elicited currents were filtered at 0.5 kHz and sampled at 2 kHz using a four-pole low-pass Bessel filter. Leak subtraction was performed using a −P/4 protocol. K_V_1.1–1.6 currents were evoked by 250-ms depolarization to 0 mV followed by a 250-ms pulse to −50 mV from a holding potential of −90 mV.

The values of I_K_ were plotted as function of voltage and fitted using the Boltzmann equation:
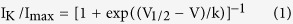
where I_max_ represents maximal I_K_, V_1/2_ is the voltage corresponding to half-maximal current, and k is the slope factor. To assess the concentration dependency of the toxin-induced inhibitory effects, a concentration-response curve was constructed, in which the percentage of current inhibition was plotted as a function of toxin concentration. Data were fitted with the Hill equation:
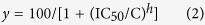
where *y* is the amplitude of the toxin-induced effect, IC_50_ is the toxin concentration at half-maximal efficacy, C is the toxin concentration, and *h* is the Hill coefficient.

Comparison of two sample means was made using a paired Student’s *t* test (p < 0.05). All data represent at least 3 independent experiments (n ≥ 3) and are presented as means ± S.E.

### Microscopy

Measurements on spheroplasts were performed using a confocal laser scanning microscope LSM 710 (Zeiss) with an αPlan-Apochromat oil immersion objective (×63, NA 1.46). Fluorescence of eGFP-OSK1 or RFP-AgTx2 was excited at the 488 or 543.5 nm wavelength and registered within the 493–600 or 560–660 nm range, respectively.

Quantitative analysis of FP-Tx binding to spheroplasts was performed as described elsewhere^21^,^22^. Briefly, an average fluorescence intensity of eGFP-OSK1 or RFP-AgTx2 associated with KcsA-K_V_1.1 or KcsA-K_V_1.3 on the spheroplast membrane was estimated for each scanned cell (as a measure of relative amount of complexes) and averaged over 150–200 cells giving the I_av_ value and its standard deviation. Dependence of I_av_ on the concentration of added FP-Tx (saturation binding curve) was measured to estimate the dissociation constant (K_d_) values. Titrations of KcsA-K_V_-bearing spheroplasts with FP-Tx were performed at a concentration of cells (and, therefore, KcsA-K_V_ receptors) ensuring the condition of [L]≫[R], where [L] and [R] are concentrations of FP-Tx and KcsA-K_V_, respectively. Accordingly, for the analysis of saturation binding curves the following equation was applied:
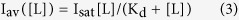
where I_sat_ is equal to the I_av_ value at the plateau.

Apparent dissociation constants (K_ap_) of non-labeled K_V_ pore blockers were estimated from the competition binding experiments. Competitions between FP-Tx and non-labeled blockers were measured when the concentration of free FP-Tx was much higher than that of the bound FP-Tx, and analyzed with the equation:
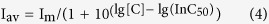
where [C] is a concentration of an added non-labeled blocker, I_m_ is I_av_ at [C] = 0, and InC_50_ is the concentration of the non-labeled blocker that displaces 50% of FP-Tx from the complex with KcsA-K_V_. The estimated InC_50_ values were used to calculate the apparent dissociation constants (K_ap_) of non-labeled blockers using the Cheng-Prusoff equation:
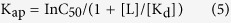


Calculated K_d_ and K_ap_ values were averaged over three independent experiments and presented as means ± S.E.

## Additional Information

**How to cite this article**: Kuzmenkov, A. I. *et al*. Fluorescent protein-scorpion toxin chimera is a convenient molecular tool for studies of potassium channels. *Sci. Rep.* **6**, 33314; doi: 10.1038/srep33314 (2016).

## Supplementary Material

Supplementary Information

## Figures and Tables

**Table 1:** IC_50_ values for toxins and FP-Tx chimeras against K_V_1 channels and corresponding Hill coefficients (*h*).

**Ligand**	**Channel**					
	**K**_**V**_**1.1**		**K**_**V**_**1.3**		**K**_**V**_**1.6**	
	**IC**_**50**_**, nM**	***h***	**IC**_**50**_**, nM**	***h***	**IC**_**50**_**, nM**	***h***
OSK1	72 ± 14	0.8 ± 0.1	6 ± 2	1.1 ± 0.2	71 ± 11	0.9 ± 0.1
eGFP-OSK1	90 ± 17	0.9 ± 0.1	8 ± 2	1.0 ± 0.1	66 ± 5	1.1 ± 0.1
AgTx2	2 ± 0.1	1.0 ± 0.1	9 ± 2	1.2 ± 0.2	92 ± 10	1.3 ± 0.2
RFP-AgTx2	3 ± 0.4	0.9 ± 0.1	83 ± 13	1.2 ± 0.2	94 ± 16	1.0 ± 0.1
