# Supplementary material for: Fluorescent protein-scorpion toxin chimera is a convenient molecular tool for studies of potassium channels
Source: Sci Rep. 2016 Sep 21;6:33314. doi: 10.1038/srep33314 (PMC5030662; doi:10.1038/srep33314)
Supplement: Supplementary Information [file srep33314-s1.pdf]

Supplementary Information

Fluorescent protein-scorpion toxin chimera is a convenient molecular tool for studies of potassium channels

Alexey I. Kuzmenkov, Oksana V. Nekrasova, Kseniya S. Kudryashova, Steve Peigneur, Jan Tytgat, Alexey V. Stepanov, Mikhail P. Kirpichnikov, Eugene V. Grishin, Alexey V. Feofanov & Alexander A. Vassilevski

DNA sequence of eGFP-OSK1

ATGGGCAGCAGCCATCATCATCATCATCACAGCAGCGGCCTGGTGCCGCGCGGCAG
CCATATGATGGTGAGCAAGGGCGAGGAGCTGTTCACCGGGGTGGTGCCCATCCTGG
TCGAGCTGGACGGCGACGTAAACGGCCACAAGTTCAGCGTGTCCGGCGAGGGCGAG
GGCGATGCCACCTACGGCAAGCTGACCCTGAAGTTCATCTGCACCACCGGCAAGCT
GCCCGTGCCCTGGCCCACCCTCGTGACCACCCTGACCTACGGCGTGCAGTGCTTCAG
CCGCTACCCCGACCACATGAAGCAGCACGACTTCTTCAAGTCCGCCATGCCCGAAG
GCTACGTCCAGGAGCGCACCATCTTCTTCAAGGACGACGGCAACTACAAGACCCGC
GCCGAGGTGAAGTTCGAGGGCGACACCCTGGTGAACCGCATCGAGCTGAAGGGCAT
CGACTTCAAGGAGGACGGCAACATCCTGGGGCACAAGCTGGAGTACAACTACAACA
GCCACAACGTCTATATCATGGCCGACAAGCAGAAGAACGGCATCAAGGTGAACTTC
AAGATCCGCCACAACATCGAGGACGGCAGCGTGCAGCTCGCCGACCACTACCAGCA
GAACACCCCCATCGGCGACGGCCCCGTGCTGTTGCCCGACAACCACTACCTGAGCA
CCCAGTCCGCCCTGAGCAAAGACCCCAACGAGAAGCGCGATCACATGGTCCTGCTG
GAGTTCGTGACCGCCGCCGGGATCACTCTCGGCATGGACGAGCTGTACGAGGGAGG
CGGTGGCTCGGGAGGTGGCGGTTCGGGCGGTGGCGGCTCCGGGGTGATAATAAATG
TGAAGTGTAAAATTTCTCGTCAATGTTTGGAACCATGCAAGAAGGCTGGAATGAGA
TTTGGAAAATGCATGAATGGCAAATGCCATTGCACACCTAAG**TGA**ATTC

Protein sequence of eGFP-OSK1

MGSSHHHHHHSSGLVP**R**GSHMMVSKGEELFTGVVPILVELDGDVNGHKFSVSGEGEGD
ATYGKLTLKFICTTGKLPVPWPTLVTTLTYGVQCFSRYPDHMKQHDFFKSAMPEGYVQ
ERTIFFKDDGNYKTRAEVKFEGDTLVNRIELKGIDFKEDGNILGHKLEYNYNSHNVYIM
ADKQKNGIKVNFKIRHNIEDGSVQLADHYQQNTPIGDGPVLLPDNHYLSTQSALSKDPN
EKRDHMVLLEFVTAAGITLGMDELYEGGGGSGGGGSGGGGSGVIINVKCKISRQCLEPC
KKAGMRFGKCMNGKCHCTPK

DNA sequence of RFP-AgTx2

ATGGTGTCTAAGGGCGAAGAGCTGATTAAGGAGAACATGCACATGAAGCTGTACAT
GGAGGGCACCGTGAACAACCACCACTTCAAGTGCACATCCGAGGGCGAAGGCAAGC
CCTACGAGGGCACCCAGACCATGAGAATCAAGGTGGTCGAGGGCGGCCCTCTCCCC
TTCGCCTTCGACATCCTGGCTACCAGCTTCATGTACGGCAGCAGAACCTTCATCAAC
CACACCCAGGGCATCCCCGACTTCTTTAAGCAGTCCTTCCCTGAGGGCTTCACATGG
GAGAGAGTCACCACATACGAAGACGGGGGCGTGCTGACCGCTACCCAGGACACCAG
CCTCCAGGACGGCTGCCTCATCTACAACGTCAAGATCAGAGGGGTGAACTTCCCATC
CAACGGCCCTGTGATGCAGAAGAAAACACTCGGCTGGGAGGCCAACACCGAGATGC
TGTACCCCGCTGACGGCGGCCTGGAAGGCAGAAGCGACATGGCCCTGAAGCTCGTG
GGCGGGGGCCACCTGATCTGCAACTTCAAGACCACATACAGATCCAAGAAACCCGC
TAAGAACCTCAAGATGCCCGGCGTCTACTATGTGGACCACAGACTGGAAAGAATCA
AGGAGGCCGACAAAGAGACCTACGTCGAGCAGCACGAGGTGGCTGTGGCCAGATA
CTGCGACCTCCCTAGCAAACTGGGGCACAAACTGAATTCTGGTTCTGGTTCTGGCCA
CATGCACCATCATCATCATCATTCTTCTGGTCTGGTGCCACGCGGTTCTGGTATGAA
AGAAACCGCTGCTGCTAAATTCGAACGCCAGCACATGGACAGCCCAGGTACCGAAA
ACCTGTATTTTCAGGGCGTGCCGATTAACGTGAGCTGTACCGGCAGCCCGCAGTGCA
TTAAACCGTGTAAAGATGCGGGCATGCGCTTTGGCAAATGCATGAACCGCAAATGT
CATTGCACCCCGAAA**T**AAGCTT

Protein sequence of RFP-AgTx2

MVSKGEELIKENMHMKLYMEGTVNNHHFKCTSEGEGKPYEGTQTMRIKVVEGGPLPF
AFDILATSFMYGSRTFINHTQGIPDFFKQSFPEGFTWERVTTYEDGGVLTATQDTSLQDG
CLIYNVKIRGVNFPSNGPVMQKKTLGWEANTEMLYPADGGLEGRSDMALKLVGGGHLI
CNFKTTYRSKKPAKNLKMPGVYYVDHRLERIKEADKETYVEQHEVAVARYCDLPSKL
GHKLNSGSGSGHMHHHHHHSSGLVPRGSGMKETAAAKFERQHMDSPGT**ENLYFQ**GVP
INVSCTGSPQCIKPCKDAGMRFGKCMNRKCHCTPK

Supplementary Tables

Suppl. Table 1. Oligonucleotides used in eGFP-OSK1 and RFP-AgTx2 gene synthesis.

**Amplification of the eGFP module**	
eGFP-f	5'-ATATCATATGATGGTGAGCAAGGGCGAG-3'
eGFP-r	5'-CCCGAACCGCCACCTCCCGAGCCACCGCCTCCCTCGTACAGCTCGTCCAT-3'
eGFP-s	5'-ATCGAATTCACTCGTACAGCTCGTCCATG-3'
**Amplification of the OSK1 module**	
OSK1-f	5'-CGGGAGGTGGCGGTTCGGGCGGTGGCGGCTCCGGGGTGATAATAAATGTG-3'
OSK1-r	5'-CGAATTCACTTAGGTGTGCAATGGCATT-3'
**Amplification of RFP module**	
RFP-f	5'-TCTCCCATGGTGTCTAAGGGCGAAGA-3'
RFP-r	5'-TCTCGAATTCAGTTTGTGCCCCAGTTTGCTAG-3'
**Amplification of the Linker module**	
L-f	5'-TCTCGAATTCTGGTTCTGGTTCTGGCCATATG-3'
L-r	5'-CTCTAAGCTTAAGGGGTACCTGGGCTGTCCATGTGCTG-3'
**Amplification of the TEV-AgTx2 module**	
AgTx-f1	5'-TCTCGGTACCGAAAACCTGTATTTTCAGGGCGTGCCGATTAACGTGAGCTGTAC-3'
AgTx-f2	5'-GTGCATTAAACCGTGTAAAGATGCGGGCATGCGCTTTGGCAAATGCATGAAC-3'
AgTx-r1	5'-TCTCAAGCTTATTTCGGGGTGCAATGACATTTGCGGTTCATGCATTTGCCAAAGC-3'
AgTx-r2	5'-CTTTACACGGTTTAATGCACTGCGGGCTGCCGGTACAGCTCACGTTAATCGGC-3'
AgTx-f1	5'- TCTCGGTACCGAAAACCTGTATTTTCAGGGCGTGCCGATTAACGTGAGCTGTAC-3'
AgTx-f2	5'- GTGCATTAAACCGTGTAAAGATGCGGGCATGCGCTTTGGCAAATGCATGAAC-3'

Suppl. Table 2. Apparent dissociation constants (K_{ap})* of complexes between pore blockers and hybrid channels that were measured in competitive binding experiments using RFP-AgTx2 and eGFP-OSK1 as labeled ligands

Ligand	**K_{ap}(KcsA-Kv1.1), nM**		**K_{ap}(KcsA-Kv1.3), nM**	
	vs. RFP-AgTx2	vs. eGFP-OSK1	vs. RFP-AgTx2	vs. eGFP-OSK1
AgTx2	0.049±0.014	0.067±0.012	0.031±0.006	0.03±0.02
OSK1	1.31±0.33	1.42±0.52	0.9±0.3	0.7±0.3
TEA	$(5.7\pm0.6)\times10^6$	$(6.5\pm1.8)\times10^6$	$(9\pm3)\times10^6$	$(6.1\pm1.8)\times10^6$

*Mean ± S.E. values are shown (n = 3).

Supplementary Figure Legends

Suppl. Fig. 1. (**a, b**) Chimeric gene structure of eGFP-OSK1 and RFP-AgTx2 cloned into pET-28a (**a**) and pET-23d (**b**). Fragments encoding FP and Tx modules and the His-tag are colored. Start and stop codons as well as restriction sites are marked. Fragments encoding the Glu-C and TEV protease cleavage sites are also shown. (**c**) Model of eGFP-OSK1 structure presenting the functional elements in different colors.

Suppl. Fig. 2. (**a**) Purification of recombinant eGFP-OSK1 chimera using size-exclusion chromatography on a TSK column. SDS-PAGE analysis is shown in the inset. Lane 1, molecular mass markers, the corresponding mass values are labeled in kDa on the left-hand side; lane 2, whole-cell lysate before IPTG treatment; lane 3, expression was induced with 0.5 mM IPTG; lane 4, affinity-purified protein; lane 5, protein after size-exclusion chromatography. (**b**) Separation of eGFP-OSK1 chimera fragments after cleavage at a glutamic acid residue using reversed-phase HPLC on a Vydac C_{18} column in a linear gradient of acetonitrile (dashed line). Molecular masses of undigested protein and fragments are shown.

Supplementary figures

Suppl. Fig. 1

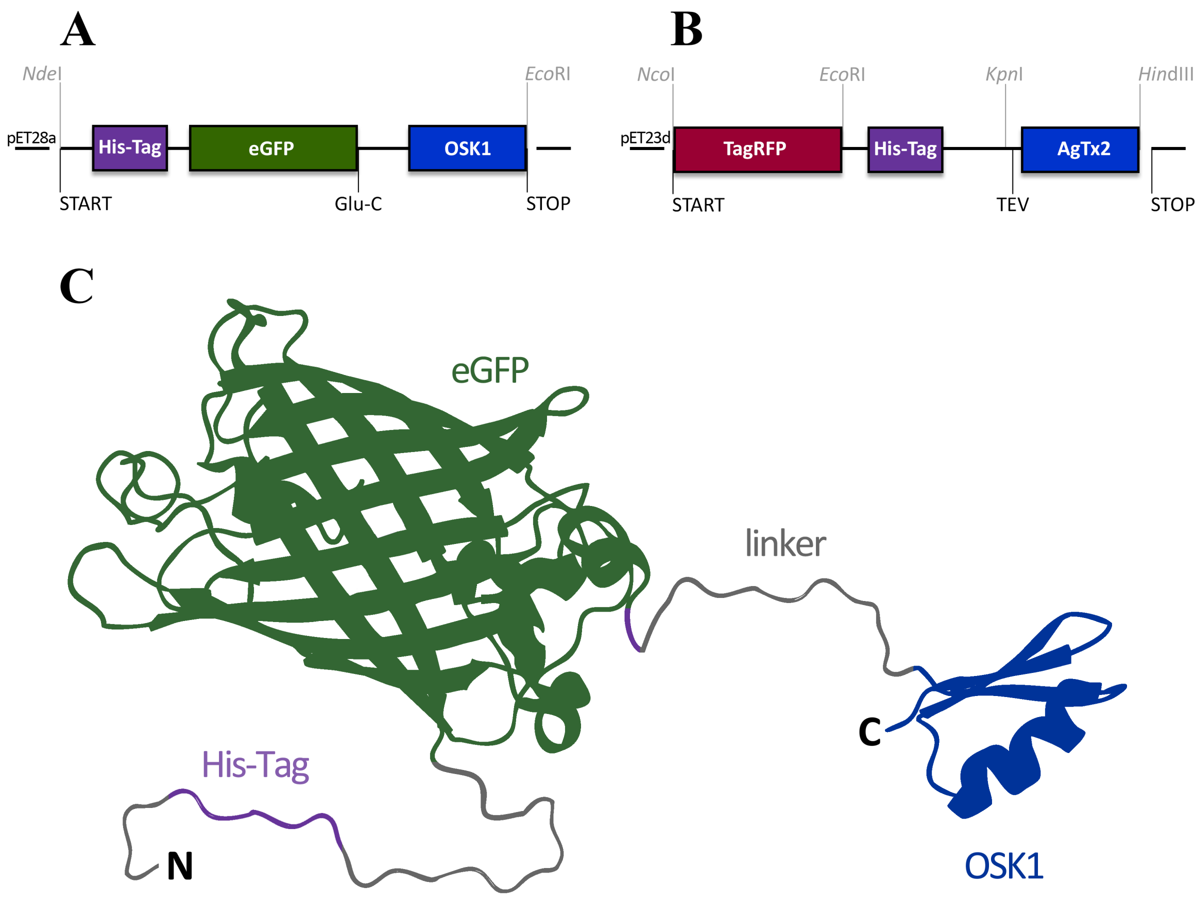

Suppl. Fig. 2

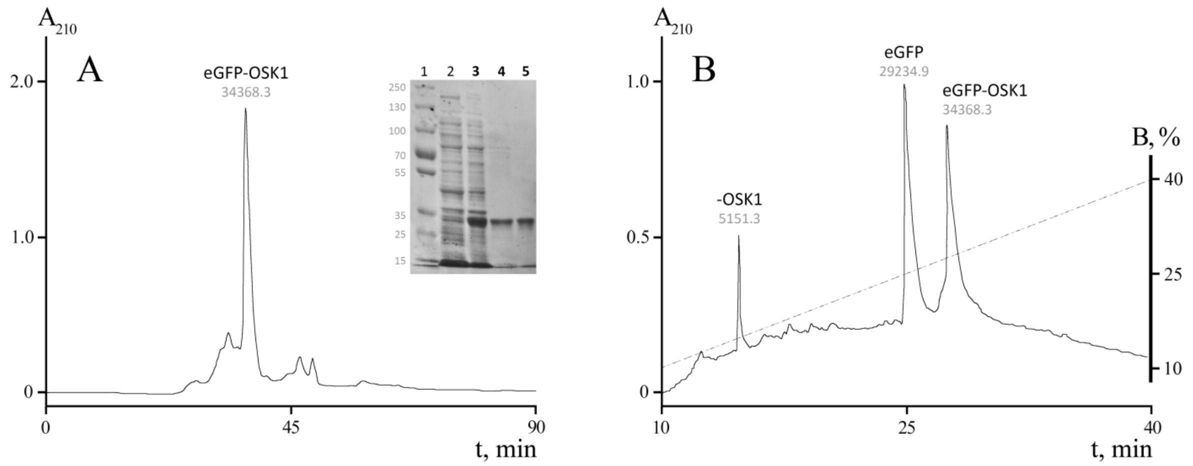
A
A210
eGFP-OSK1
34368.3
1 2 3 4 5
250
130
100
70
55
35
25
15
2.0
1.0
0
0
45
90
t, min
B
eGFP
29234.9
eGFP-OSK1
34368.3
-OSK1
5151.3
B, %
1.0
0.5
0
40
25
10
10
25
40
t, min
